# L-Methylfolate in Antidepressant Non-responders: The Impact of Body Weight and Inflammation

**DOI:** 10.3389/fpsyt.2022.840116

**Published:** 2022-03-17

**Authors:** Matthew Macaluso

**Affiliations:** Department of Psychiatry and Behavioral Neurobiology, School of Medicine, University of Alabama at Birmingham, Birmingham, AL, United States

**Keywords:** depression, inflammation, obesity, L-methylfolate, adjunctive, treatment, antidepressant, non-response

## Abstract

This summary provides context for the role of L-methylfolate (LMF) in treating antidepressant non-responders. Bidirectional relationships have been observed between obesity and/or inflammation and depression. Studies have shown an increased prevalence of depression among patients with elevated body mass index and/or chronic inflammation and an increased risk of becoming obese and experiencing chronic inflammation in those with depression. These relationships can negatively affect the pathophysiology of depression. Elevated cytokine levels have been found to be among the factors that correlate with poor antidepressant treatment responsiveness. Low baseline neurotransmitter levels (e.g., serotonin) can also be associated with reduced effectiveness of commonly used antidepressants [e.g., selective serotonin reuptake inhibitors (SSRIs)]. LMF is an approved nutritional adjunctive antidepressant therapy that increases central neurotransmitter levels and thereby improves the effectiveness of antidepressant therapy. LMF can increase clinical response when used adjunctively in patients with major depressive disorder (MDD) and who are SSRI-resistant. In 2 randomized controlled trials, the pooled results showed increased response rates (32.3 vs. 14.6%; *P* = 0.04) as measured by a ≥50% reduction or final score ≤ 7 on the Hamilton Depression Rating Scale (HAM-D) and greater mean HAM-D reductions (−5.6 vs. −3.0; *P* = 0.05) when LMF was added to an SSRI compared with an SSRI plus placebo. Additionally, LMF has demonstrated effectiveness in real-world studies, with 67.9% of patients responding to therapy, using the 9-item Patient Health Questionnaire (*P* < 0.001). *Post-hoc* analyses found that patients with inflammation and/or obesity responded better to adjunctive LMF therapy compared with the overall sample (mean HAM-D reduction: −2.74 vs. +0.99).

## Introduction

This paper provides clinically relevant context on (1) the impact of obesity and inflammation on depression and (2) the potential role of L-methylfolate (LMF) for antidepressant non-responders who have obesity and chronic inflammation. Papakostas et al. (1) first demonstrated that depressed patients with elevated body mass index (BMI) and inflammatory biomarkers responded particularly well to adjunctive LMF treatment. In subsequent years, additional data demonstrated the impact of elevated BMI and inflammation on depression and antidepressant response (2, 3), as well as the potential for LMF to be an effective adjunctive treatment in this population (4, 5). Because of the current interest on the effect of BMI and inflammation on major depressive disorder (MDD), these data will be revisited to provide additional context, potentially opening the door for additional investigation in this area.

## Inflammation and Depression

Chronic inflammation and elevated BMI are associated with an increased risk of depression (2, 6). This relationship has been shown to be bidirectional, such that individuals who are overweight/obese have a higher risk of depression (55% increased risk of developing depression; OR 1.55, 95% CI 1.22–1.98; *P* < 0.001) and those with depression have a higher risk of becoming overweight/obese (58% increased risk of obesity; OR 1.58, 95% CI 1.33–1.87; *P* < 0.001) (7). In patients with depression, the prevalence of high (>3 mg/L) C-reactive protein (CRP) levels, a biomarker for inflammation, has been shown to be 27% (95% CI, 21–34%), whereas, 58% (95% CI, 47–69%) of patients with depression had a mildly elevated CRP level (>1 mg/L) (8). Patients with depression who have a high level of CRP are also more likely to have treatment-resistant depression, suggesting that there is a subset of patients who may show additional benefit with adjunctive therapy (9). The relationship between inflammation and depression has been explained only partially by clinical and demographic factors, suggesting that yet-to-be-understood biological factors may also be involved (10). Because peripheral cytokine levels can negatively affect response to antidepressant pharmacotherapy, establishing the levels of inflammatory biomarkers may help to determine response to antidepressant treatment (3). This effect is thought to be due to cytokines altering the metabolism of monoamines, such as serotonin (11, 12). Although levels of plasma serotonin are not statistically lower in patients with MDD compared with healthy controls (1.18 ± 0.58, vs. 1.42 ± 0.67; *P* = 0.095) (13), among patients with MDD who were treated with a selective serotonin reuptake inhibitor (SSRI), patients with higher baseline levels of plasma serotonin demonstrated a significantly higher response [measured using change in Hamilton Depression Rating Scale (HAM-D) scores] compared with those with lower baseline levels of serotonin (*P* = 0.036) (13). Reduction of neurotransmitter levels due to inflammation is also thought to contribute to the development of syndromal symptoms of depression (14). Of note, higher dietary folate intake in obese/overweight women with the MTHFR C677T polymorphism, an indicator of elevated homocysteine levels and inflammation, has also been shown to reduce levels of inflammatory biomarkers, indicating that folate may play a role in the reduction of inflammation in the obese population (5).

## The Case For L-Methylfolate

LMF [Deplin; Alfasigma (Covington, LA)] is a prescription medical food approved by the US Food and Drug Administration and is indicated as an adjunctive therapy in patients with MDD who have a suboptimal level of LMF (15). LMF differs from folic acid in that it is the biologically active form of folate that more readily crosses the blood-brain barrier (16). LMF has already demonstrated efficacy as an adjunctive treatment for individuals not responding optimally to first-line antidepressant therapy (17).

The effect of LMF in patients with depression has been linked to multiple mechanisms, including monoamine synthesis, neurogenesis, and antioxidant effects (16). LMF is thought to enhance the response to antidepressant therapy due to its role in the production of tetrahydrobiopterin (BH4) and subsequent increase in monoamine synthesis (16). BH4 is a critical cofactor in the metabolic pathway for neurotransmitter synthesis and is highly labile and sensitive to oxidation (12). BH4 regulates several methylation reactions, some of which involve S-adenosylmethionine (SAMe) as a key factor, such as the metabolism of serotonin using SAMe as a methyl donor (18, 19). Therefore, reduced levels of BH4 can alter the bioavailability of neurotransmitters relevant to depression, such as serotonin and dopamine (4, 12, 18). Inflammation, through the process of oxidative stress, reduces the availability of BH4 and consequently reduces the availability of monoamines (12). Due to the role of inflammation in depleting BH4 and the resulting reduction in monoamine neurotransmitters, patients with inflammation may experience therapeutic antidepressant effects with adjunctive LMF therapy.

The effectiveness of LMF in SSRI-resistant patients with depression has been demonstrated in a number of studies. The results of 2 randomized controlled clinical trials support the use of adjunctive LMF in this patient population (17). These trials were conducted in patients with MDD who did not respond to adequate antidepressant therapy. Pooled results from both studies found that patients (patients assigned to treatment: trial 1, *n* = 148; trial 2, *n* = 75) receiving 15 mg/day of adjunctive LMF therapy showed significantly greater response rates (defined as ≥50% reduction in HAM-D score or final score ≤ 7) than those receiving an SSRI with placebo (32.3 vs. 14.6% for LMF and monotherapy, respectively; *P* = 0.04) (17). There was also a significant mean reduction in HAM-D score in patients receiving LMF 15 mg/day compared with those receiving placebo (−5.6 vs. −3.0, respectively; *P* = 0.05) (17). A 12-month open-label extension of the randomized controlled trials (N=68) found that 38% of patients receiving 15 mg/day of adjunctive LMF achieved full recovery (defined as HAM-D score ≤ 7 for at least 6 months), and no fully recovered patients experienced MDD recurrence (20). A larger, real-world, prospective observational study evaluating patients receiving LMF found that 67.9% of patients on LMF responded to treatment [defined as ≥50% reduction in the 9-item Patient Health Questionnaire (PHQ-9) score] with an average reduction of 8.5 points in the PHQ-9 score compared with baseline (*P* < 0.001) (21). Patients receiving LMF also reported: (1) a significant improvement in quality of life, with a 37% decrease in the number of patients who reported very difficult or extremely difficult quality-of-life ratings, and (2) significantly higher medication satisfaction compared with baseline (7.0 vs. 5.2, respectively, on a scale of 1 to 9; *P* < 0.001) (21).

**Figure 1:**
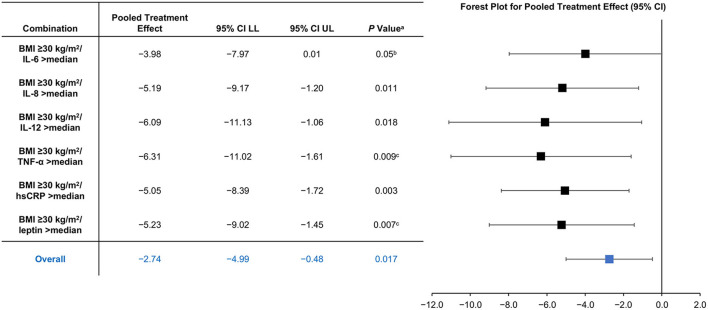
Synergistic Effects for L-Methylfolate Minus Placebo by Combination of Obesity and Inflammatory Biomarkers (22). ^a^*P*-value from χ2 test from seemingly unrelated regression. ^b^Not significant after multiple correction. ^c^*P* < 0.03 after correction for multiple testing for a priori hypothesis. BMI, body mass index; CI, confidence interval; hsCRP, high-sensitivity C-reactive protein; IL, interleukin; LL, lower limit; TNF-α, tumor necrosis factor alpha; UL, upper limit.

A *post-hoc* analysis of 2 clinical trials that evaluated adjunctive LMF therapy in those not responding to first-line antidepressant pharmacotherapy demonstrated that individuals who are obese and have high levels of inflammatory biomarker had greater reductions in HAM-D_28_ scores compared with the total sample (−2.74) and the subset of non-obese patients (+0.99) (22). It has been shown that elevated baseline levels of inflammatory biomarkers, such as tumor necrosis factor-alpha (TNF-α) and interleukin-8 (IL-8), were associated with a greater degree of treatment response to LMF (22). Combined factors, including between CRP and TNF-α as well as between BMI and IL-6 levels, suggest that there is a potentially synergistic effect of inflammatory biomarkers, obesity, and LMF treatment response (Figure 1) (22). Another *post-hoc* analysis of the same clinical trials also showed that patients with an increased BMI and high-sensitivity CRP levels showed better response (pooled mean change) than those taking placebo (1). Additionally, patients with high levels of CRP and 4-hydroxy-2-nonenal (a marker of oxidative stress) and low SAMe/S-adenosylhomocysteine ratios (a marker of methylation reactions) showed greater pooled mean change in HAM-D scores vs. placebo (−3.61, 95% CI −7.23 to 0.002, *P* = 0.05; −4.55, 95% CI −7.61 to −1.50, *P* = 0.003; −4.57, 95% CI −7.73 to −1.41, *P* = 0.005) (Table 1) (1). Contrary to expectations, markers that were previously thought to be indicative of the success of adjunctive LMF treatment, such as MTHFR polymorphisms (which can alter the metabolism of dietary folate and folic acid to LMF), did not demonstrate a profound effect. These results suggest that overweight/obese individuals with chronic low-grade inflammation not responding to first-line antidepressant therapy may respond particularly well to adjunctive LMF treatment. Although the results of these analyses are promising, randomized controlled trials among this patient population are necessary to confirm this hypothesis.

**Table 1:** Pooled effect size of L-Methylfolate by levels of biomarkers (1).

**Biomarker**	**N**	**Pooled mean change vs. placebo^**a**^**	**95% Confidence interval**	***P* Value**	**Pooled^**a**^ effect size^**b**^**
**SAM/SAH ratio**					
≥2.71	36	0.07	−3.33 to 3.48	0.966	0.01
<2.71	37	−4.57	−7.73 to −1.41	0.005	−0.75
**hsCRP**					
≥2.25 mg/L	37	−3.61	−7.23 to 0.002	0.050	−0.50
<2.25 mg/L	36	−2.29	−5.47 to 0.89	0.158	−0.36
**4-HNE**					
≥3.28 μg/mL	37	−4.55	−7.61 to −1.50	0.003	−0.74
<3.28 μg/mL	36	−0.11	−3.67 to 3.46	0.953	0.01

*^a^Pooled across study phases with equal weights. ^b^Negative pooled size indicates that the treatment effect favored the L-methylfolate group. 4-HNE, 4-hydroxy-2-nonenal; HAM-D, Hamilton Depression Rating Scale; hsCRP, high-sensitivity C-reactive protein; SAH, S-adenosylhomocysteine; SAM, S-adenosylmethionine*.

## Conclusion

In summary, the presence of inflammation and/or obesity in patients with depression can have a negative impact on the pathophysiology of depression and the effectiveness of antidepressant treatment. LMF, an effective and approved adjunctive treatment for patients with MDD who do not respond optimally to antidepressant monotherapy, has been shown to increase the effectiveness of antidepressant therapy, particularly among patients who are obese or have elevated levels of inflammatory biomarker. The proposed mechanisms of this effect include the role of inflammation in depleting BH4 and LMF's unique mechanism of action, which enhances the production of BH4, thereby promoting neurotransmitter synthesis. Although the results presented here are promising, additional randomized controlled trials should be conducted in patients with obesity/inflammation to confirm this hypothesis.

## Data Availability Statement

The original contributions presented in the study are included in the article/supplementary materials; further inquiries can be directed to the corresponding author.

## Author Contributions

MM: data analysis, interpretation, critical revision, review of the manuscript, and approval of final draft for submission.

## Funding

This study/publication received funding from Alfasigma USA, Inc. The funder was not involved in the interpretation of data, the writing of this article, or the decision to submit it for publication.

## Conflict of Interest

This study/publication received funding from Alfasigma USA, Inc. The funder was not involved in the interpretation of data, the writing of this article or the decision to submit it for publication. The author declares that the research was conducted in the absence of any commercial or financial relationships that could be construed as a potential conflict of interest.

## Publisher's Note

All claims expressed in this article are solely those of the authors and do not necessarily represent those of their affiliated organizations, or those of the publisher, the editors and the reviewers. Any product that may be evaluated in this article, or claim that may be made by its manufacturer, is not guaranteed or endorsed by the publisher.
